# Oxacillinase-484–Producing Enterobacterales, France, 2018–2023

**DOI:** 10.3201/eid3010.240814

**Published:** 2024-10

**Authors:** Cécile Emeraud, Sandrine Bernabeu, Delphine Girlich, Inès Rezzoug, Agnès B. Jousset, Aurélien Birer, Thierry Naas, Rémy A. Bonnin, Laurent Dortet

**Affiliations:** Bicêtre Hospital, Le Kremlin-Bicetre, France (C. Emeraud, S. Bernabeu, I. Rezzoug, A.B. Jousset, T. Naas, R.A. Bonnin, L. Dortet);; INSERM, Paris, France (D. Girlich);; Centre National de Référence de la Résistance aux Antibiotiques, Clermont-Ferrand, France (A. Birer)

**Keywords:** Antimicrobial resistance, oxacillinase, OXA-484, Enterobacterales, France, carbapenemase, *Escherichia coli*, *E. coli* ST410, *E. coli* ST1722, epidemiology, bacteria

## Abstract

We examined the emergence and characteristics of oxacillinase-484–producing Enterobacterales in France during 2012–2023. Genomic analysis identified 2 predominant sequence types in *Escherichia coli*: ST410 and ST1722. Plasmid analysis revealed that *bla*_OXA-484_ genes were carried mostly on an IncX3-type plasmid associated with genetic elements including insertion sequences IS*3000* and IS*Kpn19*.

Carbapenemase-producing Enterobacterales (CPEs) pose a considerable threat to public health because of antimicrobial resistance. In France, the most prevalent carbapenemases are OXA-48–like type. More than 55 variants of OXA-48–like enzymes have been identified (*1*); OXA-48, OXA-181, and OXA-244 are most prevalent, but OXA-484 has been increasingly identified. OXA-484 differs from OXA-48 by 5 amino acid substitutions (Thr104Ala, Asn110Asp, Glu168Gln, Ser171Ala, Arg214Gly) and from OXA-181 by a single amino acid at position 214 within the β5-β6 loop (*2*). Most prior reports have described OXA-484 in *Escherichia coli* (*3*–*6*), but some reports also have identified it in *K. pneumoniae* (*2*,*7*), *K. aerogenes* (*6*), and *K. variicola* (*8*). Reported OXA-484–producing *E. coli* was noted to belong mainly to the sequence type (ST) 410 (*3*,*5*,*6*), and *bla*_OXA-484_ genes were carried mainly 51-kb IncX-type plasmids (*3*,*4*,*6*). Given the similar genetic background of *bla*_OXA-484_ and *bla*_OXA-181_, several studies suggested that *bla*_OXA-484_ could directly derive from *bla*_OXA-181_ (*3*,*8*). We used whole-genome sequencing to decipher the epidemiology of OXA-484–producing Enterobacterales in France during 2012–2023.

## The Study

**Figure 1:**
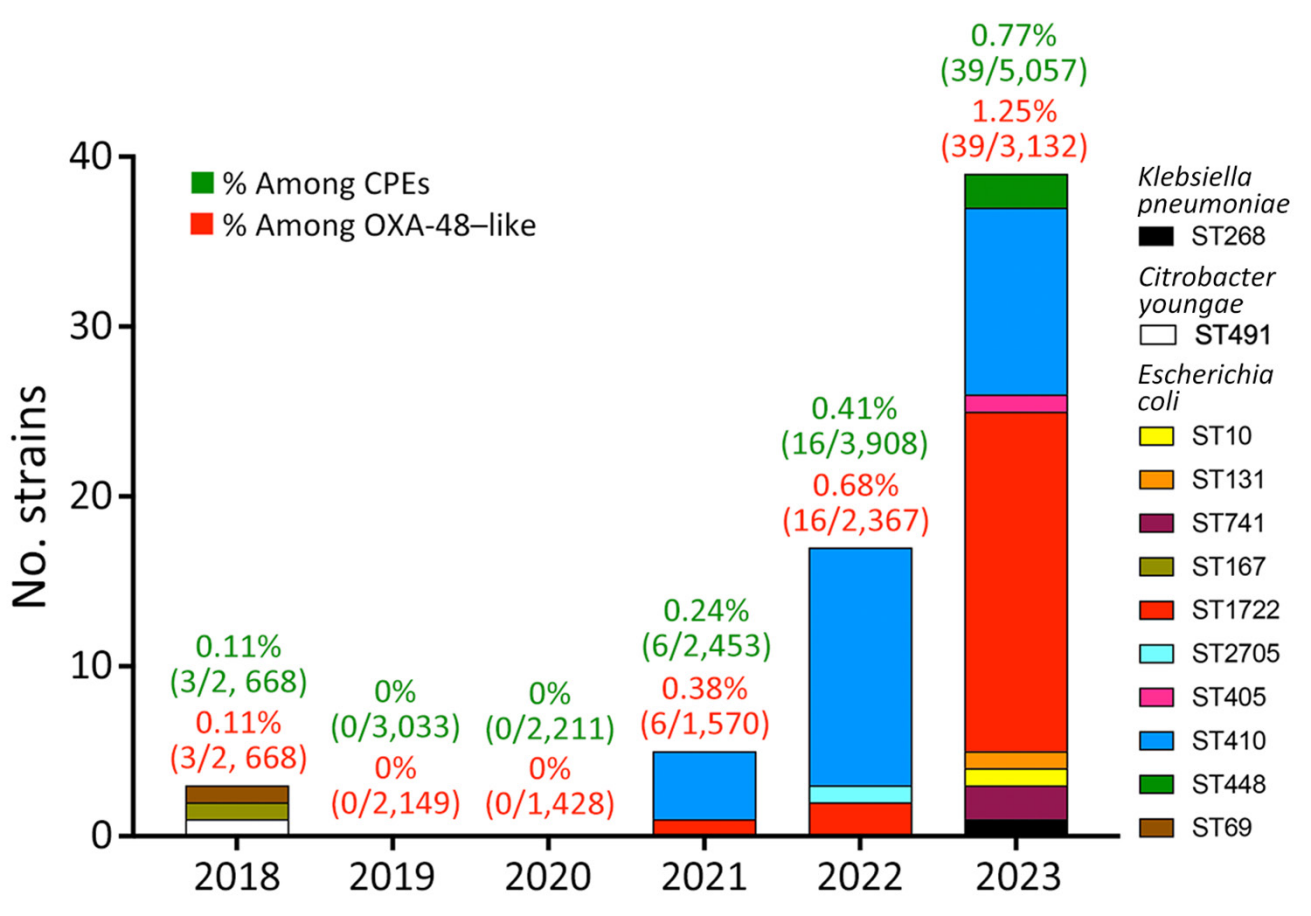
Evolution of number of OXA-484–producing CPE isolates received at the French National Reference Center for Carbapenem-Resistant Enterobacterales, France, 2012–2023. No OXA-484–producing isolate was received before 2018. Colors indicate species and STs. CPE, carbapenem-resistant Enterobacterales; OXA, oxacillinase; ST, sequence type.

The French National Reference Center (Le Kremlin-Bicêtre, France) receives bacterial strains sent by microbiology laboratories throughout France to analyze for suspected carbapenemase production. The center received 64 clinical, nonduplicate OXA-484–producing isolates during 2012–2023 (Appendix 1 Table 1). We identified OXA-484 producers in 3 isolates from 2018 (0.11% of CPEs, 0.16% of OXA-48–like), 6 from 2021 (0.24% of CPEs, 0.38% of OXA-48–like), 16 from 2022 (0.41% of CPEs, 0.68% of OXA-48–like), and 39 from 2023 (0.77% of CPEs, 1.24% of OXA-48–like) (Figure 1). All OXA-484 producers were *E. coli*, except 1 *K. pneumoniae* and 1 *Citrobacter youngae*. Short-read next-generation sequencing (Appendix 2) showed that the *C. youngae* isolate belonged to ST491 and the *K. pneumoniae* isolate to ST268. We identified 10 single STs among the 62 *E. coli* isolates; most were ST410 (n = 29) and ST1722 (n = 23).

**Figure 2:**
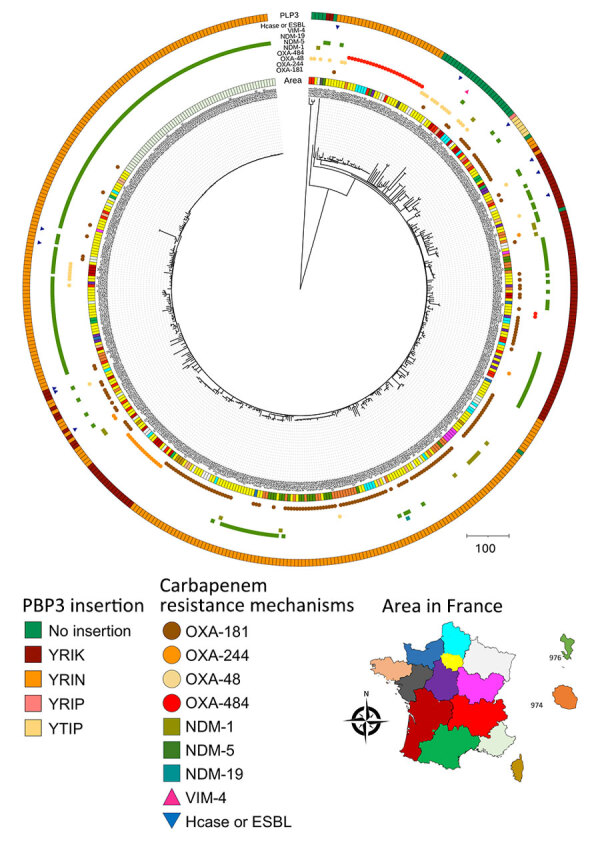
Phylogenetic tree and location of carbapenem-resistant *Escherichia coli* sequence type (ST) 410 isolates received at the French National Reference Center for Carbapenem-Resistant Enterobacterales, France, 2012–2023. Carbapenemases are classified into 3 of the 4 Ambler classes: class A (mainly *Klebsiella pneumoniae* carbapenemase); class B or metallo-β-lactamases (predominantly NDM, VIM, or imipenemase types); and class D, primarily OXA-48–like types, including OXA-484 producers. Carbapenemase types are detailed and OXA-484 producers are indicated. ST410 strains are distinguished by whether there are amino acid insertions (YRIK, YRIN, YRIP, or YTIP motifs) in PBP3. We conducted the single-nucleotide polymorphism analysis on a common genome representing 89.05% of the entire genome of the reference strain 303D1. Map shows locations where specific mechanisms were found. ESBL, extended-spectrum β-lactamase; Hcase, helicase; NDM, New Delhi metallo-β-lactamase; OXA, oxacillinase; PBP3, penicillin-binding protein 3; VIM, Verona integron-encoded metallo-β-lactamase.

To compare strains belonging to the 2 predominant sequence types (ST410 and ST1722), we constructed a matrix of single-nucleotide polymorphisms (SNPs) in SNIppy version 4.6.0 (ILRI-CGIAR, https://hpc.ilri.cgiar.org). We found 2 ST410 strains (399A8 and 415H3), isolated in the same region, were notably distinct from the others (>1,000 SNPs with the remaining strains) (Appendix 2 Figure 1, panel A). For the other strains, all isolated from different areas, we observed a maximum of 57 SNPs between any 2 strains. We constructed a phylogenetic tree that included all *E. coli* ST410 strains received at the F-NRC (n = 459). Except for 399A8 and 415H3, the OXA-484 producers formed a distinct cluster among *E. coli* ST410 (Figure 2).

We observed a maximum of 35 SNPs among the OXA-484–producing *E. coli* ST1722 (Appendix 2 Figure 1, panel B); all OXA-484 producers clustered inside *E. coli* ST1722 (n = 43) (Appendix 2 Figure 2). Mapping of short-read data by using CLC Genomics Workbench (QIAGEN, https://www.qiagen.com) onto previously published plasmids (GenBank accession nos. NZ_OP594535, CP058621, CP076530, OP594534, NZ_JANQAU010000004, and CP076530) revealed a high nucleotide identity in most strains. In *E. coli*, plasmids harboring *bla*_OXA-484_ appeared similar to the IncX3 described by Moser et al*.* (*3*), with the exception of 2 strains belonging to ST1722, in which plasmids were closed to the IncFIA-like/IncFIB-like/IncFII-type plasmid reported by Findlay (*5*) (Appendix 1 Table 2). In addition, in *C. youngae* and *E. coli* 172D10 strains, the *bla*_OXA-484_ gene was located on a new 58,440 pb IncP1 plasmid. For strains having short reads that did not map accurately to described plasmids (query cover <90%, n = 10; Appendix 1 Table 2), we performed long-read sequencing as previously described (*9*) to reconstruct these plasmids using MinION technology (Oxford Nanopore Technologies, https://nanoporetech.com). The sizes of *bla*_OXA-484_–carrying plasmids were 18,731–162,425 bp; 7/10 possessed replicases close to colKP3-like, IncFIA-like, IncFIB-like, and IncFII, and 3 contained only colKP3-like. Some plasmids carried other resistance genes, notably *qnrS1* (9/10 plasmids) and *mph(A)* (4/10 plasmids) (Appendix 1 Table 3).

**Figure 3:**
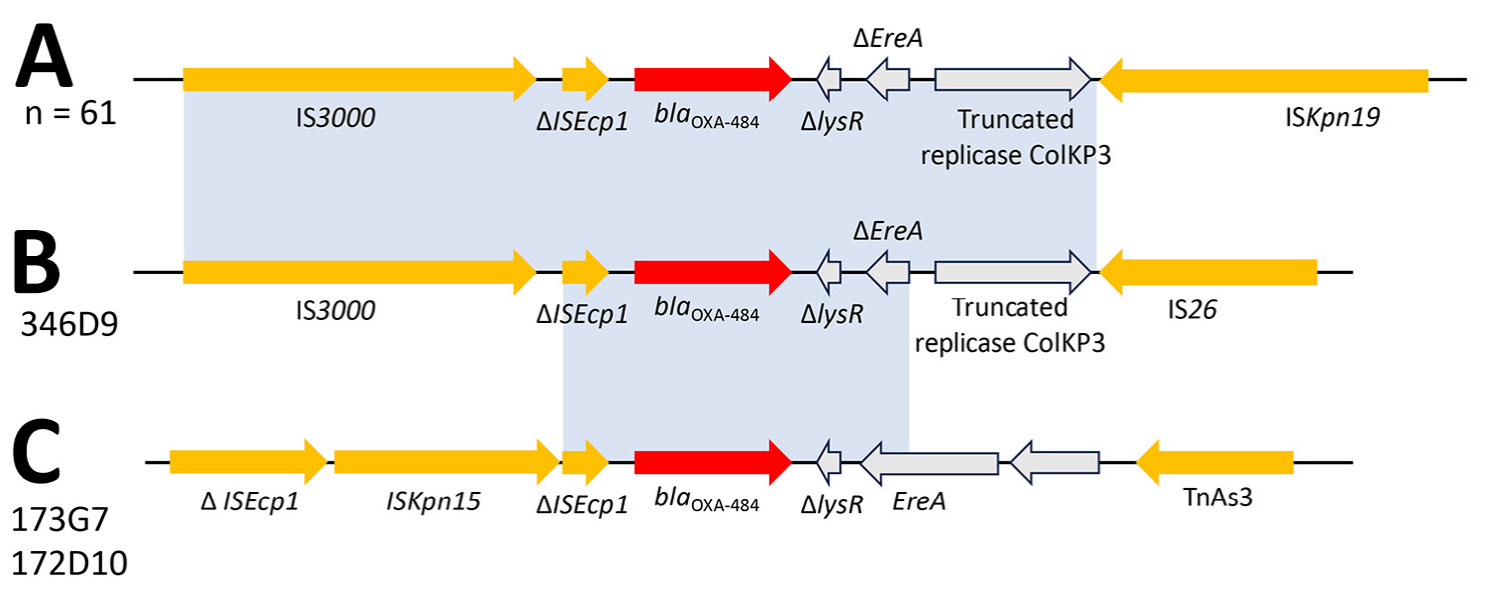
Genetic environments of *bla*_OXA-484_ genes for 64 strains included in a study of oxacillinase-484–producing isolates received at the French National Reference Center for Carbapenem-Resistant Enterobacterales, France, 2012–2023. A) All strains; B) strain 346D9; C) strains 173G7 and 172D10. The *bla*_OXA-484_ genes are shown in red, mobile elements are in yellow, and other genes in gray. The sequence homologies represented in light blue show an identity of >99.95%.

We analyzed the close genetic context of *bla*_OXA-484_ and found the same genetic environment for 62/64 strains (Figure 3). For strain 346D9, the close genetic environment was slightly smaller, with the IS*3000* upstream and the truncated ColKP3 replicase downstream and an IS*26* instead of the IS*Kpn19*. For the *bla*_OXA-484_ carried by the IncP1-type plasmid, the genetic environment was totally different, with various truncated IS elements (Figure 3). We found no homologous sequences associated with OXA-48–like or any other carbapenemase-encoding gene.

We determined MICs of various antimicrobial drugs for the entire collection (Table; Appendix 1 Table 4). As expected, OXA-484 producers exhibited low MICs for the 3 carbapenems and were susceptible to imipenem/relebactam and meropenem/vaborbactam combinations (with no inhibitory role of relebactam and vaborbactam). Given that most OXA-484 producers coproduce an extended-spectrum – β-lactamase (CTX-M-14 or CTX-M-15) or an acquired cephalosporinase (mostly CMY-42), those we analyzed were found to be resistant to third- and fourth-generation cephalosporins and to aztreonam, but they remained susceptible to ceftazidime/avibactam and aztreonam/avibactam. All strains were susceptible to colistin (Table). 

**Table:** Antimicrobial susceptibility of OXA-484-producing isolates received at the French National Reference Center for Carbapenem-resistant Enterobacterales, 2018–2023*

Species and carbapenemase type	MICs for antimicrobial drugs												
	ERT	IMI	IMI/ REL	MEM	MEM/VAB	CPM	CTZ	CTZ/ AVI	CFT/ TAZ	AZT	AZT/ AVI	TMC	COL
*Escherichia coli* OXA-484													
None, n = 26													
MIC	≤0.25–8	≤0.25–1	≤0.25–1	≤0.25–2	≤0.25–2	≤1–2	≤0.25–8	≤0.25–1	0.5–>8	≤0.25–2	≤0.25–2	32–>128	0.25–1
MIC_50_	0.5	≤0.25	≤0.25	≤0.25	≤0.25	≤1	≤0.25	≤0.25	2	≤0.25	≤0.25	64	0.5
MIC_90_	1	0.5	0.5	0.5	0.5	≤1	2	0.5	>8	1	1	>128	1
ESBL, n = 5													
MIC	≤0.25–1	≤0.25	≤0.25	≤0.25	≤0.25	2–>16	1–>16	≤0.25–0.5	1–>8	2–>16	≤0.25–1	32–128	0.25–0.5
MIC_50_	0.5	≤0.25	≤0.25	≤0.25	≤0.25	16	4	0.5	8	>16	≤0.25	32	0.25
MIC_90_	1	≤0.25	≤0.25	≤0.25	≤0.25	>16	>16	0.5	>8	>16	1	128	0.5
Acquired, n = 26													
MIC	0.5–4	≤0.25–2	≤0.25–1	≤0.25–2	≤0.25–1	≤1–16	4–>16	≤0.25–4	1–>8	1–>16	≤0.25–8	32–>128	0.5
MIC_50_	1	≤0.25	≤0.25	≤0.25	≤0.25	8	>16	2	>8	>16	2	>128	0.5
MIC_90_	4	0.5	0.5	0.5	0.5	4	>16	4	>8	>16	4	>128	0.5
*E. coli* OXA-484 + NDM													
ESBL, n = 2,	>16	8–>8	8–>8	>16	>16	>16	>16	>16	>8	>16	1–2	>128	0.5
Acquired, n = 2,	2–4	>8	>8	>16	>16	>16	>16	>16	>8	>16	4–2	>128	0.5
*E. coli* OXA-484 + OXA-48, acquired, n = 1	0.5	2	1	4	2	8	1	0.5	>8	4	2	32	0.5
*Citrobacter youngae* OXA-484, acquired, n = 1	2	0.5	0.5	0.5	0.5	1	4	≤0.25	>8	1	≤0.25	>128	0.5
*Klebsiella pneumoniae* OXA-484, n = 1	1	≤0.25	≤0.25	0.5	≤0.25	≤1	≤0.25	≤0.25	1	≤0.25	≤0.25	128	0.5
Total, n = 64													
MIC_50_	2	≤0.25	≤0.25	≤0.25	≤0.25	4	8	0.5	>8	16	1	128	0.5
MIC_90_	4	1	1	2	1	>16	>16	2	>8	>16	4	>128	0.5
% Susceptible†	31.3	95.3	93.8	92.2	93.8	42.2	39.1	93.8	34.4	35.9	54.7	0	100

*AVI, avibactam; AZT, aztreonam; CFT, ceftolozane; COL, colistin; CPM, cefepime; CTZ, ceftazidime; ESBL, extended-spectrum β-lactamase; IMI, imipenem; ERT, ertapenem; MEM, meropenemNDM, New Delhi metallo-β-lactamase; ; OXA, oxacillinase; REL, relebactam; TAZ, tazobactam; TMC, temocillin; VAB, vaborbactam. †Interpreted according to EUCAST guidelines.

ST410 *E. coli* strains possess a 4-aa insertion in penicillin-binding protein 3 (e.g., YRIN motif) (*10*) that confers decreased susceptibility to certain antibiotics (e.g., ceftazidime and aztreonam). In our collection, all OXA-484–producing ST410 *E. coli* strains possessed the YRIN insertion in penicillin-binding protein 3 (Figure 2) and exhibited higher MICs for aztreonam/avibactam and ceftazidime/avibactam compared with strains belonging to other sequence types (Appendix 2 Figure 3). In contrast, strains belonging to ST1722 exhibited very low MICs.

A finding of low MICs of temocillin in OXA-484 producers compared with OXA-48 producers aligned with previous reports regarding OXA-244 producers (*11*) that exhibited the same Arg214Gly mutation within the β5–β6 loop. Because increased susceptibility to temocillin was responsible for screening issues of OXA-244 (*11*), we evaluated the accuracy of several screening media to detect OXA-484 producers. We selected 46 OXA-484 producers with varying temocillin MICs alongside control strains producing OXA-48 or other carbapenemases. We streaked the strains with 10 µL of 0.5 McFarland bacterial suspension onto 2 commercially available CPE screening media (ChromID CARBA SMART [bioMérieux, https://www.biomerieux.com] and mSuperCARBA [Mast Diagnostic, https://www.mast-group.com/fr]). As expected, after 16–24 hours’ incubation, only 2 strains cultured on the CARB-side and only 11 on the OXA-side of the ChromID CARBA SMART medium. Those 11 isolates displayed temocillin MICs ≥128 mg/L. The mSuperCARBA agar yielded positive results for all OXA-484 producers.

## Conclusions

As previously reported for OXA-244 producers (*11*), we found that the prevalence of OXA-484 producers is likely underestimated because of detection failure related to a lack of sensitivity of ChromID CARBA SMART, the most common screening medium in France. Whereas mSuperCARBA agar appears to be well suited, this medium possesses lower specificity, leading to a rise in additional confirmatory tests (*12*). 

Most previously reported OXA-484 producers belong to *E. coli* *ST410*, a widely distributed, high-risk clone (*13*) that usually exhibits reduced susceptibility to multiple antimicrobial drugs, complicating treatment options. Given the number of SNPs between all OXA-484–producing *E. coli* ST410 isolates of our collection and their geographic distribution, those strains do not originate from a single outbreak. However, OXA-484–producing *E. coli* ST410 strains clustered together, suggesting the circulation of a single clone in France during 2018–2023. In addition, most OXA-484–producing *E. coli* belonged to ST1722. This clone has not been recognized for its prevalence nor for its role in the spread of carbapenem resistance but has been associated with the production of extended-spectrum β-lactamases in France (*14*).

We found *bla*_OXA-484_ genes localized on different plasmids but with a common close genetic environment characterized by IS*3000* upstream and IS*Kpn19* downstream. The ST410 *E. coli* carried *bla*_OXA-484_ on a 51-kb IncX3 plasmid similar to the one described by Moser et al. (*4*), whereas the ST1722 *E. coli* carried it on an IncF plasmid similar to the one described by Findlay et al (*6*). The genetic environment of *bla*_OXA-484_ described on IncX3 or IncF-type plasmids is close to that of *bla*_OXA-181_, likely the result of a mutation in *bla*_OXA-181_ (*8*). However, the genetic environment observed around the *bla*_OXA-484_ gene located on the IncP1-type plasmid does not match any known structures of other *bla*_OXA-48_–like genes, questioning its potential origin. In summary, the growing prevalence of OXA-484 producers highlight the urgent need for improved surveillance of these pathogens.

Appendix 1 Genomic sequence data from a study of oxacillinase-484–producing Enterobacterales, France, 2018–2023.

Appendix 2 Additional information on oxacillinase-484–producing Enterobacterales, France, 2018–2023.
