# Supplementary material for: Oxacillinase-484–Producing Enterobacterales, France, 2018–2023
Source: Emerg Infect Dis. 2024 Oct;30(10):2178–82. doi: 10.3201/eid3010.240814 (PMC11431933; doi:10.3201/eid3010.240814)
Supplement: Appendix 2 — Additional information on oxacillinase-484–producing Enterobacterales, France, 2018–2023. [file 24-0814-Techapp-s2.pdf]

Article DOI: http://doi.org/10.3201/eid3010.240814

EID cannot ensure accessibility for supplementary materials supplied by authors. Readers who have difficulty accessing supplementary content should contact the authors for assistance.

Oxacillinase-484-Producing Enterobacterales, France, 2018–2023

Appendix 2

Additional Methods

We conducted short-read next-generation sequencing on the entire set of 64 OXA-484–producing strains by using HiSeq technology (Illumina, htttps:///www.illumina.com; BioProject accession no. PRJNA1119420). We assembled reads by using Shovill version 1.1.0 (https://github.com/tseemann/shovill) and SPAdes version 3.14.0 (https://github.com/ablab/spades). We carried out multilocus sequence typing and resistome analyses by using the pubMLST (https://pubmlst.org) and Resfinder 4.5.0 (http://genepi.food.dtu.dk/resfinder) databases.

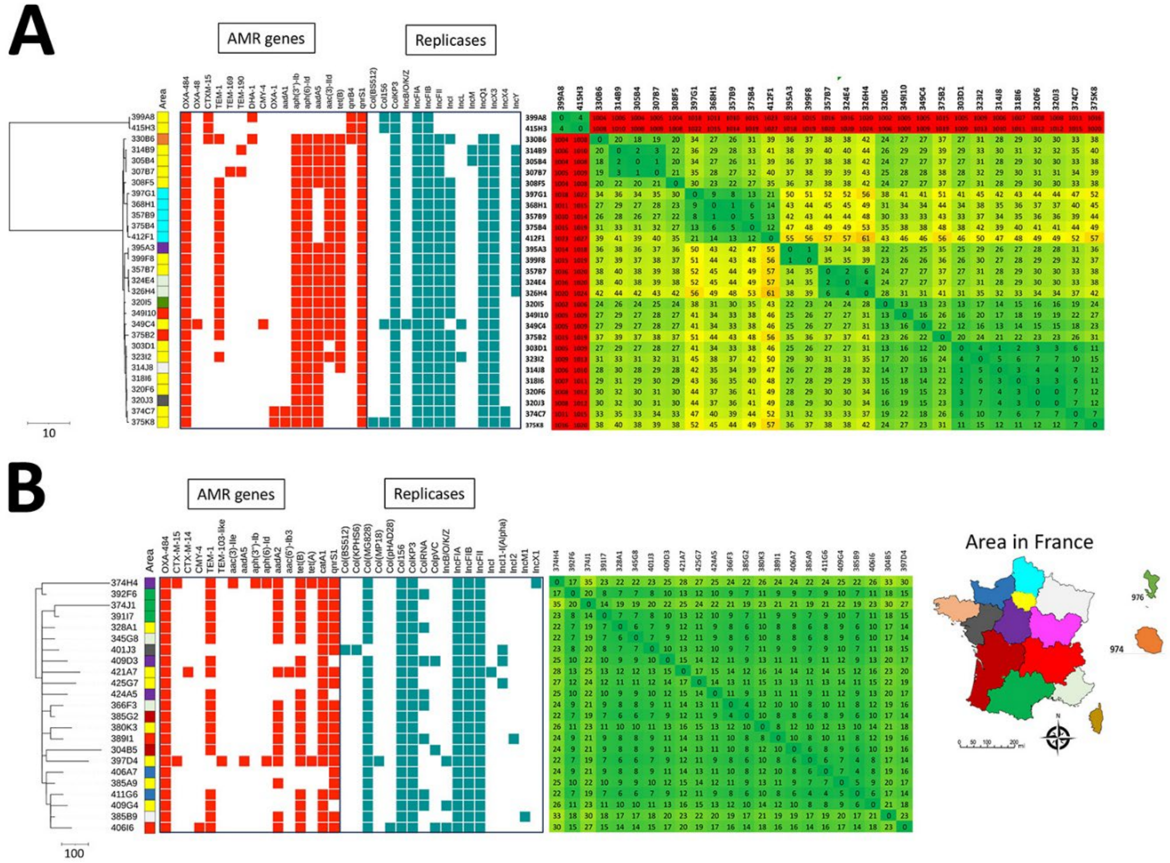

Appendix 2 Figure 1. Phylogenetic tree with SNPs matrix (single-nucleotide polymorphism) and global characterization (multilocus sequence typing, plasmid, and β-lactamase content) for OXA-484–producing *Escherichia coli* belonging to ST410 (A) and ST1722 (B).

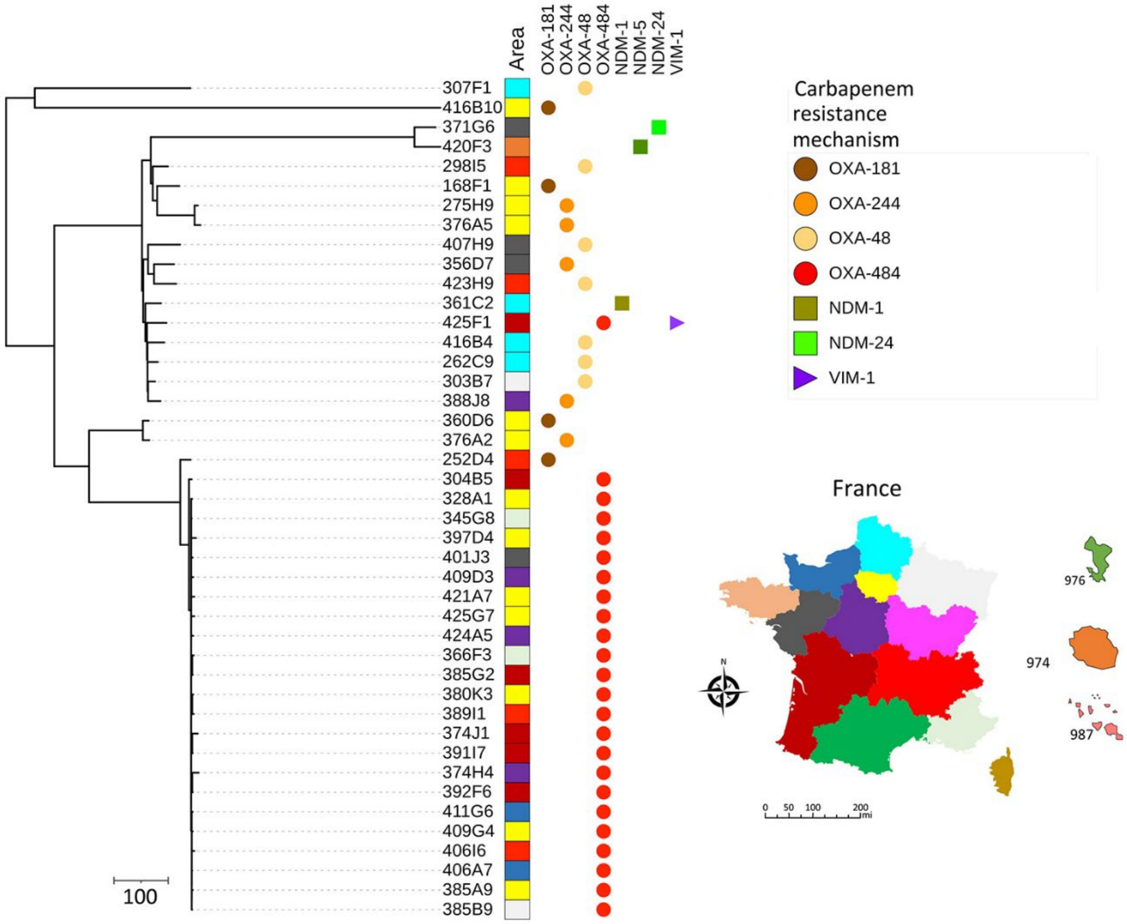

Appendix 2 Figure 2. Phylogenetic tree of ST1722 isolates received at the FNR-C in France from 2012 to 2023. Carbapenemase types are detailed in this figure and OXA-484 producers are indicated with red circles. ST410 strains are indicated by whether there are amino acid insertions (YRIK, YRIN, YRIP, or YTIP motifs) in penicillin-binding-protein 3. We conducted the single-nucleotide polymorphism analysis on a common genome representing 90.15% by using the reference strain 304B5.

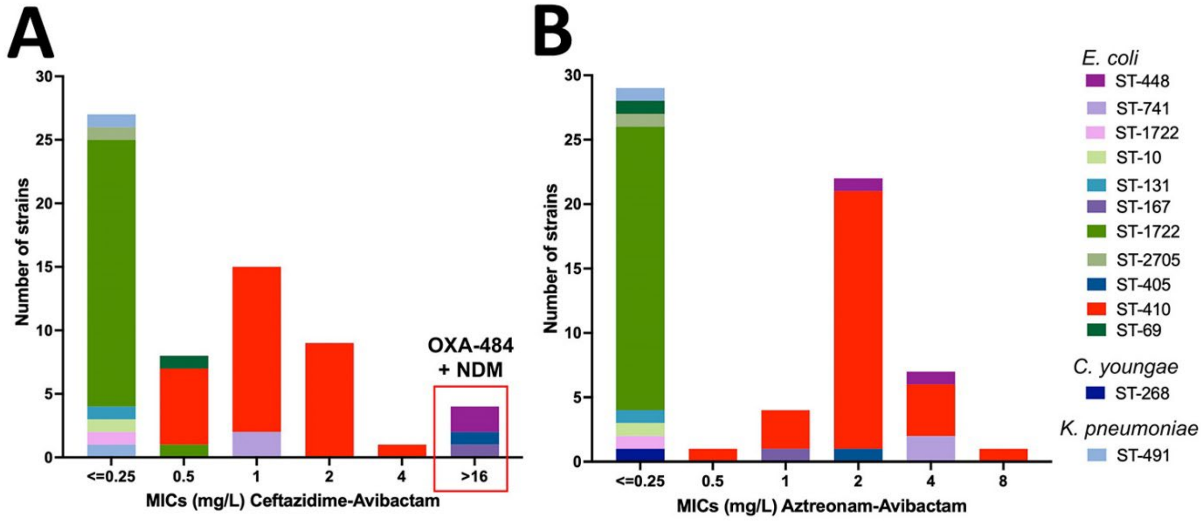

Appendix 2 Figure 3. Distribution of minimal inhibitory concentrations (MICs) for ceftazidime-avibactam and aztreonam-avibactam of OXA-484–producing isolates according to their sequence type. NDM, New Delhi metallo-β-lactamase.
